# Transcriptomic analysis reveals the early body wall regeneration mechanism of the sea cucumber *Holothuria leucospilota* after artificially induced transverse fission

**DOI:** 10.1186/s12864-023-09808-1

**Published:** 2023-12-12

**Authors:** Renhui Liu, Xinyue Ren, Junyan Wang, Ting Chen, Xinyu Sun, Tiehao Lin, Jiasheng Huang, Zhengyan Guo, Ling Luo, Chunhua Ren, Peng Luo, Chaoqun Hu, Xudong Cao, Aifen Yan, Lihong Yuan

**Affiliations:** 1 School of Life Sciences and Biopharmaceutics, Guangdong Pharmaceutical University, Guangzhou, 510006 People’s Republic of China; 2 School of Medicine, Foshan University, Foshan, 528000 People’s Republic of China; 3 Key Laboratory of Breeding Biotechnology and Sustainable Aquaculture, Key Laboratory of Tropical Marine Bio-resources and Ecology (LMB), South China Sea Institute of Oceanology, Chinese Academy of Sciences, Guangzhou, 510301 People’s Republic of China; 4 Guangdong Institute for Drug Control, Guangzhou, 510301 People’s Republic of China; 5 Guangxi Key Laboratory of Marine Environmental Science, Guangxi Beibu Gulf Marine Research Center, Guangxi Academy of Sciences, Nanning, 530007 People’s Republic of China; 6 Department of Chemical and Biological Engineering, University of Ottawa, Ottawa, ON 999040 Canada

**Keywords:** Transcriptome, Body wall, Regeneration, Transverse fission, Sea cucumber

## Abstract

**Background:**

Sea cucumbers exhibit a remarkable ability to regenerate damaged or lost tissues and organs, making them an outstanding model system for investigating processes and mechanisms of regeneration. They can also reproduce asexually by transverse fission, whereby the anterior and posterior bodies can regenerate independently. Despite the recent focus on intestinal regeneration, the molecular mechanisms underlying body wall regeneration in sea cucumbers still remain unclear.

**Results:**

In this study, transverse fission was induced in the tropical sea cucumber, *Holothuria leucospilota*, through constrainment using rubber bands. Histological examination revealed the degradation and loosening of collagen fibers on day-3, followed by increased density but disorganization of the connective tissue on day-7 of regeneration. An Illumina transcriptome analysis was performed on the *H. leucospilota* at 0-, 3- and 7-days after artificially induced fission. The differential expression genes were classified and enriched by GO terms and KEGG database, respectively. An upregulation of genes associated with extracellular matrix remodeling was observed, while a downregulation of pluripotency factors *Myc*, *Klf2* and *Oct1* was detected, although *Sox2* showed an upregulation in expression. In addition, this study also identified progressively declining expression of transcription factors in the Wnt, Hippo, TGF-β, and MAPK signaling pathways. Moreover, changes in genes related to development, stress response, apoptosis, and cytoskeleton formation were observed. The localization of the related genes was further confirmed through in situ hybridization.

**Conclusion:**

The early regeneration of *H. leucospilota* body wall is associated with the degradation and subsequent reconstruction of the extracellular matrix. Pluripotency factors participate in the regenerative process. Multiple transcription factors involved in regulating cell proliferation were found to be gradually downregulated, indicating reduced cell proliferation. Moreover, genes related to development, stress response, apoptosis, and cell cytoskeleton formation were also involved in this process. Overall, this study provides new insights into the mechanisms of whole-body regeneration and uncover potential cross-species regenerative-related genes.

## Background

Regeneration, a widespread phenomenon in nature, refers to the precise process of reconstructing the injured or lost parts of an organism or its organs [1]. In terms of regenerative capabilities, invertebrates commonly surpass vertebrates [2]. Specially, certain invertebrates like sea cucumbers and sea stars utilize regeneration as a means of asexual reproduction [3, 4]. Nevertheless, despite extensive research, the mechanism behind regeneration remains incompletely comprehended and exhibits variations across diverse species. For example, flatworms possess the astonishing capability to regenerate their entire bodies from minute fragments [5]. Salamanders, on the other hand, can regenerate not only complete limbs and tails but also complicated organs like eyes, nerve and heart [6, 7]. Vertebrates like zebrafish exhibit the ability to regenerate fins following injuries [8], while human livers have demonstrated a regenerative capacity after sustaining damage [9]. Invertebrates such as echinoderms also showcase impressive regenerative prowess, with the ability to regenerate injured or lost organs, as seen in sea urchins [10], sea stars [11] and sea cucumbers [12].

Sea cucumbers, in particular, serve as an exceptional model organism for the study of regeneration. When faced with stressful situations, they have the remarkable ability to expel certain internal organs and subsequently regenerate the lost ones [12]. Recent studies regarding sea cucumber regeneration have primarily focused on the renewal process of diverse organs, including the intestinal tract [13, 14], respiratory tree [15], body wall [16], and tube foot [17]. The body wall tissue of a sea cucumber comprises an epithelial layer, connective tissue layer, muscle layer, and coelomic epithelial layer [16]. Despite extensive observation of sea cucumber body wall regeneration at the level of cellular histology [16], the molecular mechanisms underlying this regenerative process remain unclear.

The sea cucumber *Holothuria glaberrima* has been widely utilized as a model for investigating regeneration processes [13]. Cellular events involved in *H. glaberrima* intestinal regeneration encompass an increase in spherule-containing cells, remodeling of the extracellular matrix, formation of spindle-like structures, and robust cellular division, primarily occurring in the coelomic epithelium [16]. During the early stages of intestinal regeneration, there was a significant upregulation in the transcriptional activities of genes, as supported by a transcriptomic analysis [18]. Damage triggers a strong stress response, even during late stages of regeneration, including increased levels of reactive oxygen species (ROSs), activation of antioxidant enzymes, immune system components, and the involvement of extracellular matrix (ECM) remodeling and Wnt signaling [12]. For the pluripotency factors, also known as Yamanaka factors, *SoxB1*, *Myc* and *Bmi-1* are expressed in the regeneration processes of *H. glaberrima* radial nerve cord and the digestive tube but lack a coordinated regulation way [19, 20]. Furthermore, differential expression of numerous genes involved in development, ECM formation, and cytoskeletal construction was observed during intestinal regeneration in the sea cucumber *Apostichopus japonica* [21].

The tropical sea cucumber *Holothuria leucospilota*, belong to phylum Echinodermata and class Holothuroidea, are primarily distributed in the Indo-Pacific region [22, 23]. *H. leucospilota* has both sexual and asexual reproduction methods, with transverse fission being the primary asexual one [24, 25]. Studies have shown that after transverse fission, *H. leucospilota* can regenerate the anterior body out of the posterior body and vice versa [26]. The artificial spawning and culture of *H. leucospilota* have been reported recently [27]. In addition, the extrinsic (death receptor-mediated) [28, 29] and the intrinsic (mitochondrial-mediated) [30, 31] mechanisms of apoptosis in *H. leucospilota* coelomocytes under pathogenic and environmental stresses have also been studied. Aim to investigate the molecular mechanism of sea cucumber early body wall regeneration, a transcriptome analysis was performed with the *H. leucospilota* at 0-, 3- and 7-days after artificially induced fission. The regenerated morphologies and histological characteristics were first clarified. In addition, the signaling pathway and differential expression genes (DEGs) related to body wall regeneration were identified. The involved genes were further determined by in situ hybridization (*IS*H). This study can provide new insights into the mechanisms of whole-body regeneration and uncover potential cross-species regenerative-related genes.

## Results

### Morphological and histological changes of the body wall during regeneration

**Fig. 1:**
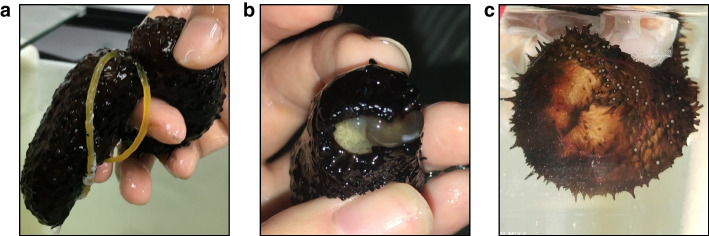
**a** Schematic diagram demonstrating the experimental procedure. The precise position for middle strangulation wound was confirmed in the sea cucumber in natural state, and subsequently, the wound was tightly secured using a leather band. **b** The wound at the head end of the sea cucumber after the occurrence of the strangulation injury was divided into two. **c** The growth of the anus at the head end of the sea cucumber was carried out 9 days following the strangulation (7 days post-wound formation)

Artificially induced transverse fission was performed on the sea cucumber *H. leucospilota* (Fig. 1a). Within 48 h after skin strangulation, all individuals underwent splitting into two parts, and the internal organs were observed through the fracture (Fig. 1b). The wound completely healed within 7 days, indicating the completion of early body wall regeneration (Fig. 1c).

**Fig. 2:**
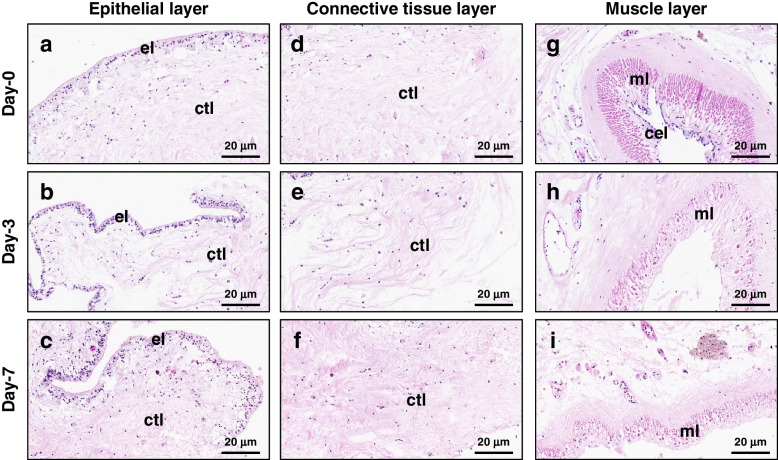
Histological sections of the body wall stained by HE. **a-c** The epithelial layer of the body wall at the wound site on day-0 (**a**), -3 (**b**), and -7 (**c**) of regeneration. **d-f** The connective tissue layer of the body wall at the wound site on day-0 (**d**), -3 (**e**), and -7 (**f**) of regeneration. **g-i** The muscle layer of the body wall on day-0 (**g**), -3 (**h**), and -7 (**i**) of regeneration. el: epithelial layer; ctl: connective tissue layer; ml: muscle layer; cel: coelomic epithelial layer

Histological examination of the wound site on day-3 and day-7 of regeneration revealed no significant changes in the epithelial layer of the body wall (Fig. 2a-c). The connective tissue collagen fibers of normal sea cucumber are closely arranged, and a few cells are scattered in the connective tissue (Fig. 2d). However, degradation and loosening of collagen fibers were observed in the connective tissue layer on day-3, resulting in its laxity (Fig. 2e). The connective tissue appeared denser but exhibited disorganization compared to normal tissue on day-7 (Fig. 2f). Myocyte de-differentiation was evident in the vicinity of the injury site during both day-3 and day-7 of regeneration, with disorganized, attenuated, or complete absence of muscle layers or bundles observed at these two time points (Fig. 2g-i). Notably, the coelomic epithelial layer was absent within the inner part of the muscle layer during day-3 and day-7 of regeneration.

### Sequence mapping and transcript assembly

**Fig. 3:**
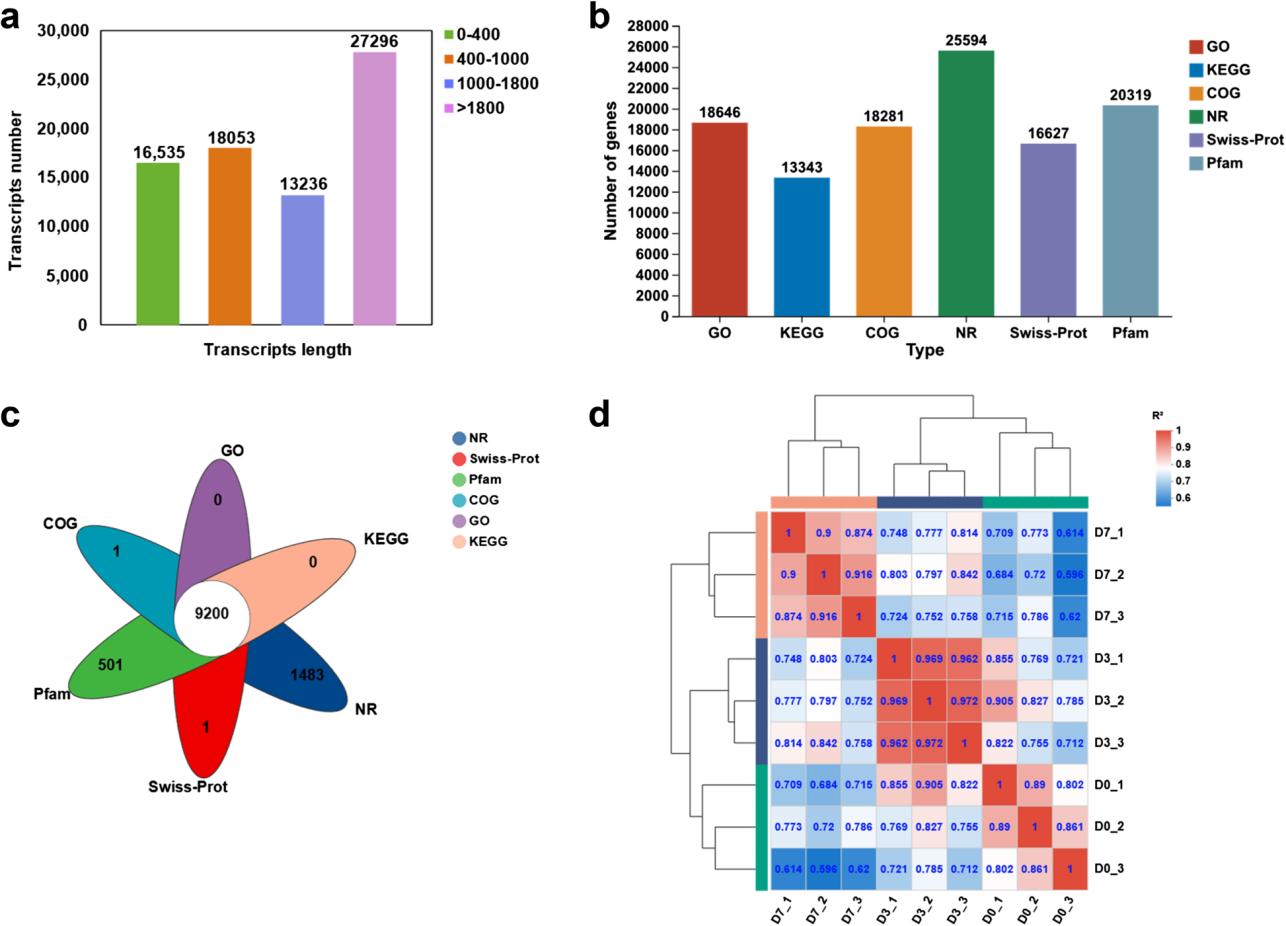
Transcriptome information and inter-sample correlation analysis. **a** Distribution profile of transcript lengths. The x-axis represents the range of transcript length, while the y-axis indicates the number of transcripts within each length range. **b** Histogram demonstrating the annotation of gene functions. The x-axis denotes the name of the database, while the y-axis represents the number of sequences annotated to each respective database. **c** Venn diagram depicting the annotation of gene functions. Circles of different colors signify the number of genes annotated to various databases, with the overlapping sections indicating genes simultaneously annotated in multiple libraries. **d** Correlation analysis for the gene expression of body wall tissue samples from day-0, -3, and -7 of regeneration. The values within the graph represent correlation coefficients between the two samples, where a higher value indicates a greater similarity

After collecting the raw transcriptomic sequencing data, the clean reads were obtained by filtering them using the fastp software. The clean reads generated for day-0, day-3 and day-7 samples of body wall regeneration ranged from 41,794,852 to 58,307,640, the sequencing quality was excellent, with Q20 values exceeding 95% (Table 1). TopHat2 was employed to align the clean reads to the reference genome, resulting in unique mappings. The unique mappings for day-0, day-3 and day-7 samples ranged from 72.0% to 75.18% (Table 2). Finally, transcript assembly was performed, resulting in a total of 75,710 transcripts. The length distribution of transcripts exhibits considerable variation. The dataset consists of 16,535 transcripts within the 0–400 bp range, 18,053 transcripts within the 400–1000 bp range, and 13,326 transcripts within the 1000–1800 bp range. Notably, the largest subset comprises transcripts larger than 1800 bp, totaling 27,796 in count (Fig. 3a).

**Table 1:** Quality control and data statistics for clean reads

Sample	Raw reads	Raw bases	Clean reads	Clean bases	Q20 (%)	GC (%)
D0_1	52,232,426	7,834,863,900	51,422,422	7,633,255,120	97.06	40.84
D0_2	56,707,192	8,506,078,800	56,167,888	8,329,855,042	98.06	40.69
D0_3	54,774,270	8,216,140,500	54,236,606	8,033,593,537	97.91	40.80
D3_1	50,997,666	7,649,649,900	50,646,084	7,537,888,515	98.48	39.33
D3_2	47,079,904	7,061,985,600	46,774,762	6,959,278,275	98.49	40.00
D3_3	42,036,766	6,305,514,900	41,794,852	6,213,782,512	98.45	39.92
D7_1	56,305,090	8,445,763,500	55,922,502	8,336,197,794	97.99	42.43
D7_2	58,735,492	8,810,323,800	58,307,640	8,695,815,340	97.97	42.24
D7_3	53,688,932	8,053,339,800	53,270,318	7,952,423,632	97.96	41.87

**Table 2:** Sequence mapping analysis

Sample	Total reads	Total mapped	Multiple mapped	Uniquely mapped
D0_1	51,422,422	38,655,466 (75.17%)	1,315,741 (2.56%)	37,339,725 (72.61%)
D0_2	56,167,888	43,100,355 (76.73%)	1,346,508 (2.4%)	41,753,847 (74.34%)
D0_3	54,236,606	41,615,094 (76.73%)	1,383,151 (2.55%)	40,231,943 (74.18%)
D3_1	50,646,084	38,552,970 (76.12%)	1,213,204 (2.4%)	37,339,766 (73.73%)
D3_2	46,774,762	36,289,081 (77.58%)	1,124,163 (2.4%)	35,164,918 (75.18%)
D3_3	41,794,852	31,950,487 (76.45%)	1,076,728 (2.58%)	30,873,759 (73.87%)
D7_1	55,922,502	42,720,139 (76.39%)	1,844,842 (3.3%)	40,875,297 (73.09%)
D7_2	58,307,640	43,748,345 (75.03%)	1,651,961 (2.83%)	42,096,384 (72.2%)
D7_3	53,270,318	39,886,594 (74.88%)	1,533,830 (2.88%)	38,352,764 (72.0%)

### Functional annotation of genes

To annotate the genes, BLASTx was employed using various protein databases. The proteins with the highest sequence similarity were selected for annotation. Out of the total number of genes, 31,343 (63.08%) were successfully annotated. Specifically, among the annotated genes, 25,594, 13,343, 16,627, 18,646, 18,281 and 20,319 were annotated with the NR, KEGG, SWISS-PROT, GO, COG and PFAM databases, respectively (Fig. 3b). The Venn diagram illustrates the number of expressed genes that were annotated in different combinations of databases. A total of 9,200 genes were simultaneously annotated in multiple libraries, while 1,483 and 501 genes were exclusively annotated in the NR and PFAM databases, respectively (Fig. 3c).

### Differentially expressed genes (DEGs)

**Fig. 4:**
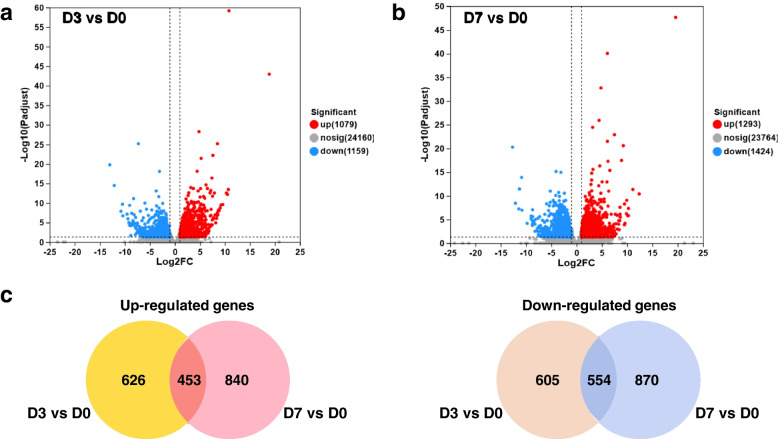
**a** & **b** Differentially expressed genes (DEGs) in the day-3 (**a**) and day-7 (**b**) compared to the day-0 regenerated groups. Volcano plots illustrating DEGs in the day-3 and day-7 groups, respectively. The x-axis represents the fold change in gene expression, while the y-axis represents the statistical test value of the gene expression variation. The values on both axes are logarithmically normalized. Each point on the plot corresponds to a specific gene; red points indicate significantly up-regulated genes, blue points indicate significantly down-regulated genes, and gray points represent genes with no significant difference. **c** Venn diagram presenting the number of commonly and significantly differentially up-regulated and down-regulated genes across different regeneration time points

By correlation analysis, a high correlation coefficient indicated a strong resemblance in transcript expression between biological replicate samples (Fig. 3d). Differentially expressed genes (DEGs) were identified as adjusted *P* < 0.05 and |log2FC |≥ 1 (FC means fold change here). Volcano plots were employed to analyze the distinctively expressed genes between the day-3 (Fig. 4a) and day-7 (Fig. 4b) groups compared to the day-0 group. In the day-3 group, a total of 2,238 significantly differentially expressed genes were observed, with 1,079 genes being up-regulated and 1,159 genes being down-regulated (Fig. 4a). Similarly, in the day-7 group, 2,717 significantly differentially expressed genes were identified, with 1,293 genes being up-regulated and 1,424 genes being down-regulated (Fig. 4b). Furthermore, a total of 1,007 genes were found to be co-expressed in both the day-3 and day-7 groups, consisting of 453 up-regulated genes and 554 down-regulated genes (Fig. 4c).

### Functional classification of DEGs

**Fig. 5:**
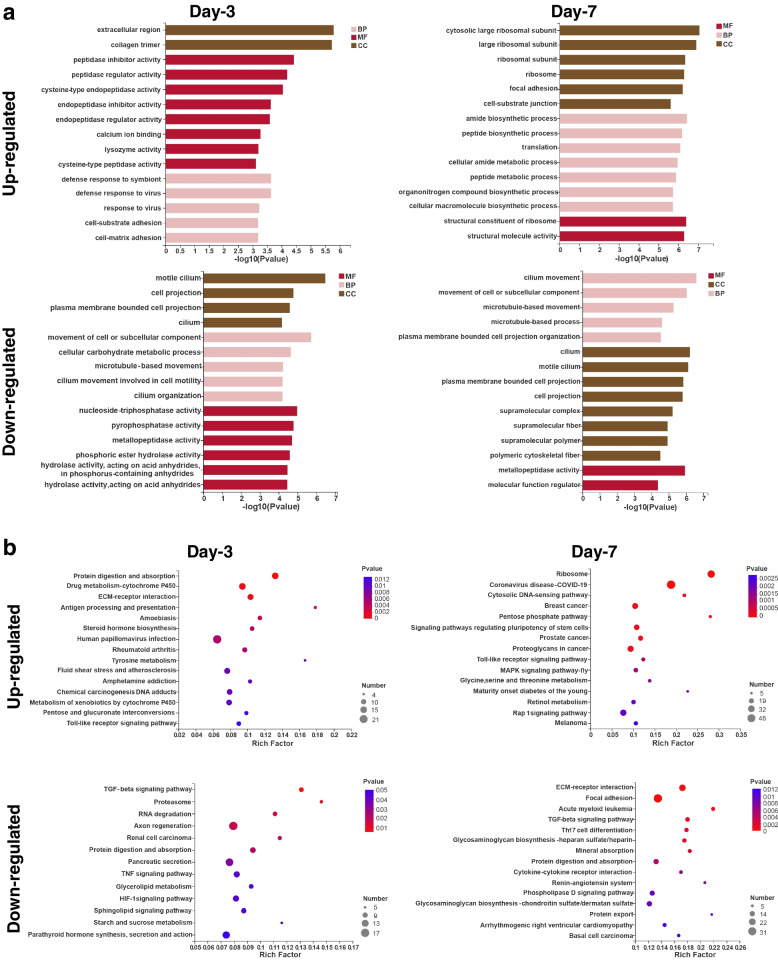
**a** GO classification results of DEGs in the day-3 and day-7 groups in comparison to the day-0 group are summarized into three main GO categories, namely cellular components (CC), molecular functions (MF), and biological processes (BP). The Y-axis represents the GO ontology, while the X-axis indicates the significance level of enrichment. **b** KEGG classification of DEGs in the day-3 and day-7 groups in comparison to the day-0 group. The y-axis represents the pathway name, while the x-axis indicates the Rich factor. A larger Rich factor suggests a more significant enrichment. The size of the bubbles corresponds to the number of genes in the pathway, and the color of the bubbles indicates different *P* value ranges. Only the top 15 enrichment results are displayed

The Gene Ontology (GO) terms derived from DEGs were exhibited in Fig. 5a. In the upregulated genes, significant enrichment was observed on day-3 for terms related to the extracellular region, collagen trimer, peptidase and endopeptidase activities, calcium ion binding, lysozyme activity and immune-related functions. On the other hand, terms associated with ribosomal subunit, ribosome, focal adhesion, cell-substrate junction, protein synthesis and metabolism significantly enriched on day-7. For downregulated genes, enrichment was observed on both days for terms related to motile cilium, cell projection, plasma membrane bounded cell projection, cilium, movement of cell or subcellular components, microtubule-based movement and metallopeptidase activity.

To further illustrate the pathways linked to body wall regeneration, the DEGs enrichment were further analyzed with the Kyoto Encyclopedia of Genes and Genomes (KEGG) database (Fig. 5b). On day-3, upregulated genes demonstrated significant enrichment in the pathways of ECM-receptor interaction, Antigen processing and presentation and Protein digestion and absorption. Conversely, on day-7, the enriched pathways among the upregulated genes were Signaling pathways regulating pluripotency of stem cells, MAPK signaling pathway-fly and Retinol metabolism. Notably, the Toll-like receptor signaling pathway exhibited significant enrichment on both days. Among the downregulated genes, there was enrichment of both the TGF beta signaling pathway and Protein digestion and absorption pathways on both days. Specifically, on day-3, the Proteasome, RNA degradation and Axon regeneration pathways displayed enrichment, while on day-7, the ECM-receptor interaction and Focal adhesion pathways were enriched.

### Expression analysis of genes related to regeneration

**Fig. 6:**
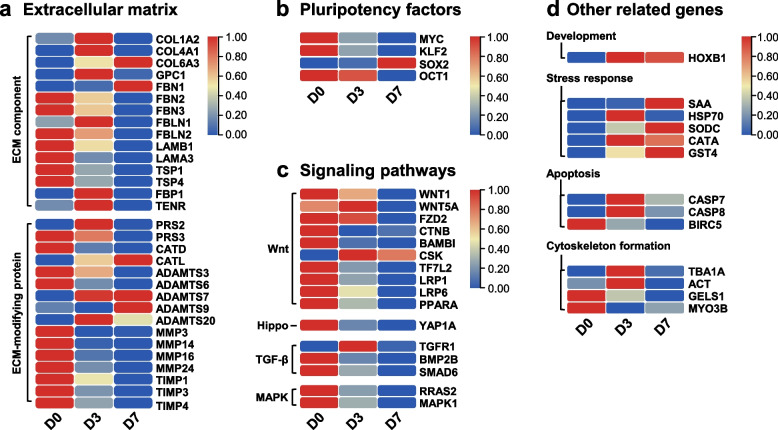
Heat map illustrating the change in expression trends of DEGs related to extracellular matrix reconstruction (**a**), pluripotency factors (**b**), signaling pathway (**c**) and other regenerated factors (**d**) during genes the non-regeneration phase (D0) and regeneration at day-3 (D3) and day-7 (D7). The genes related to extracellular matrix reconstruction include ECM component and ECM-modifying protein; the pluripotency factors include *Myc*, *Klf2*, *Sox2* and *Oct1*; the genes related to signaling pathway include Wnt, Hippo, TGF-β and MAPK; The genes related to other regenerated factors include development, stress response, apoptosis and cytoskeleton

Based on GO category and KEGG enrichment analysis, the genes crucial for body wall regeneration can be clarified into four distinctive groups: ECM-associated genes (Fig. 6a), pluripotency factors (Fig. 6b), signaling pathways (Fig. 6c) and other genes related to the process of regeneration (Fig. 6d).

Among the ECM-associated genes, several were found to be upregulated during one or multiple stages of regeneration. These genes include *COL*s, *GPC1*, *FBN1*, *PRS2*, *CATL*, *ADAMTS7*, *9* and *20*, *FBLN1* and *TENR* (Fig. 6a). In contrast, the expression levels of *FBN2* and *3*, *FBLN2*, *LAM*s, *TSP*s, *PRS3*, *CATD*, *ADAMTS3* and *6*, *MMP*s and *FBP1* were found to be downregulated during the process of regeneration (Fig. 6a).

Four Pluripotency factors were identified, namely *Myc*, *Klf2*, *Sox2* and *PO2F1* (*Oct1*), as shown in Fig. 6b. Among these factors, *Sox2* exhibited upregulation on day 7 of the regeneration, whereas the expression levels of *Myc*, *Klf2* and *Oct1* were observed to be downregulated.

As shown in Fig. 6c, the analysis revealed differential expression patterns of various transcription factors within the Wnt, Hippo, TGF-β and MAPK signaling pathways. While *Wnt5A* and *CSK* exhibited upregulation in the Wnt signaling pathway, the transcription factors *Wnt1*, *FZD2*, *CTNB*, *BAMBI*, *TF7L2*, *LRP*s and *PPARA* showed downregulation progressively. Similarly, in the Hippo signaling pathway, *YAP1A* was downregulated, whereas *BMP2B*, *SMAD6* and *BAMBI* in the TGF-β signaling pathway, as well as *RRAS2* and *MAPK1* in the MAPK signaling pathway, were found to be downregulated during the process of regeneration.

Other genes associated with the regeneration process are presented in Fig. 6d. Among these differentially expressed genes, we found the greatest difference in up-regulation of SAA and down-regulation of GELS1 during regeneration. The expression of the development-related gene *Hox-B1* was found to be upregulated, while immune-related genes such as *HSP70* and *SAA* and several antioxidant enzymes including *SODC*, *CATA* and *GST4* showed upregulation during regeneration. Conversely, the expression levels of apoptosis-related genes *CASP7* and *CASP8* were upregulated, whereas *BIRC5* exhibited downregulation. Furthermore, cytoskeleton-related genes *TBA1A* and *ACT* displayed upregulation, while *GELS1* and *MYO3B* were observed to be downregulated.

### Validation of regeneration-associated genes by *ISH*

**Fig. 7:**
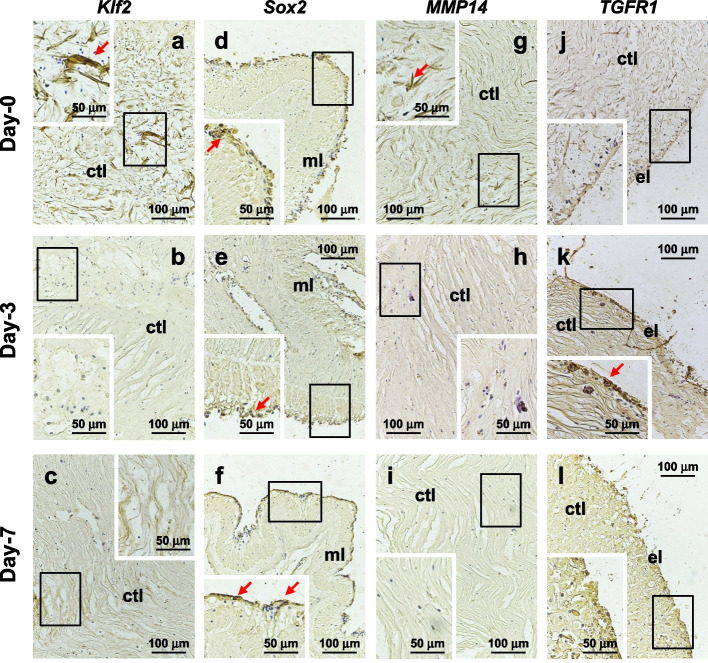
In situ hybridization (*IS*H) illustrating the spatial expression patterns of *Klf2* (**a-c**), *Sox2* (**d-f**), *MMP14* (**g-i**) and *TGFR1* (**j-l**) during the early regeneration phase of the body wall. The three rows of horizontal images represent tissue sections at 0 (**a**, **d**, **j**, **g**), 3 (**b**, **e**, **h**, **k**) and 7 (**c**, **f**, **i**, **l**) days of body wall regeneration, respectively. Insets provide a high magnification view of the boxed areas in the corresponding main micrographs. el: epithelial layer; ctl: connective tissue layer; ml: muscle layer

*IS*H was conducted to investigate the spatial and temporal distributions of regeneration-associated genes *Klf2*, *Sox2*, *MMP14* and *TGFR1* during early body wall regeneration of *H. leucospilota*. The results revealed that *Klf2* displayed extensive expression in the connective tissues of the day-0 sample, but limited positive signals were observed in the day-3 and day-7 samples (Fig. 7a-c). *MMP14* exhibited widespread expression in the connective tissues of the day-0 sample, but minimal expression in regenerating connective tissue (Fig. 7d-f), which aligns with the down-regulation trend of *Klf2* and *MMP14* indicated by the RNA sequencing data on day-3 and day-7 of regeneration. *Sox2* gene expression was notably high in the coelomic epithelial layer and the positive signal for *Sox2* intensified during the early regeneration of the somatic wall (Fig. 7g-i), supporting the RNA sequencing data indicating an increase of *Sox2* expression during early regeneration. Moreover, no positive signal for *TGFR1* was observed in the epithelial layer of the day-0 sample, but there was an increase in positive signal on day-3 followed by a decrease on day-7 (Fig. 7j-l), supporting the RNA sequencing data that demonstrated an up-regulation of *TGFR1* on day-3 and a return to normal levels on day-7.

## Discussion

Asexual reproduction has been documented in 16 holothurian species, occurring through transverse fission (architomy) and fragmentation in adult sea cucumbers [4]. Previous studies have demonstrated the ability of sea cucumbers *Cladolabes schmeltzii*, *Colochirus robustus*, *Pseudocolochirus violaceus* and *Holothuria scabra* to regenerate their anterior and posterior portions following transverse surgery [26]. Natural asexual reproduction through fission has been observed as a common phenomenon in *H. leucospilota* [32]. In this study, we present a novel method for artificially inducing transverse fission in *H. leucospilota* by gradually constraining its middle section using rubber bands (Fig. 1a). Based on this approach, Histological and transcriptomic analyses were conducted to explore the underlying mechanisms of whole-body regeneration in sea cucumbers.

The body wall of sea cucumbers primarily consists of connective tissue, which enables fission by transforming the extracellular matrix [33]. This specific connective tissue, referred to as mutable collagenous tissue (MCT) [34, 35] or catch connective tissue [36], possesses the unique capability to modify its mechanical properties [37]. The stiffness of MCT is determined by the interaction of three protein groups: matrix metalloproteinases (MMPs), tissue inhibitors of metalloproteinases (TIMPs), and cross-link complexes that interconnect collagen fibrils [38]. An increase in MMPs concentration or activity in the connective tissue leads to the degradation of cross-link complexes. This breakdown enables collagen fibrils to slide along one another, leading to the compliant pattern of MCT [12]. Consequently, local changes in the properties of the body wall’s connective tissue facilitate the sea cucumbers divided into two parts.

Sea cucumbers possess the remarkable capability of undergoing visceral evisceration and regenerating lost organs as a response to environmental deterioration [39] or various human impacts [40]. After evisceration, rapid wound healing and activation of the immune system occur [12]. The regenerated tissue layers of the intestine originate from the relevant components of the mesentery, esophageal stump, and cloacal stump [13]. Upon division, the anterior fragment contains the aquapharyngeal bulb, gonad, Polian vesicles and the front half of the intestinal tube, while the posterior fragment retains the rear gut portion, cloaca and respiratory trees [12]. Both fragments undergo wound healing, followed by the regeneration of missing structures, such as the aquapharyngeal bulb in the posterior fragment and the cloaca in the anterior fragment [4]. Additionally, the remaining intestine extends either forwards or backwards until a complete intestinal tube is formed.

The present transcriptomic analysis has revealed the differential expression of ECM-related genes during the body wall regeneration of *H. leucospilota*. Upregulated expression was observed for ECM-related genes such as *COL*s, *GPC1*, *FBN1*, *PRS2, CATL*, *ADAMTS7*, *9* and *20*, F*BLN1* and *TENR* during regeneration, while downregulated expression was observed for *FBN2* and *3*, *FBLN2*, *LAM*s, *TSP*s, *PRS3*, *CATD*, *ADAMTS3* and *6*, *MMP*s, and *FBP*1 (Fig. 6a). ECM is an essential component of connective tissues, providing physical support and regulates cellular processes [41]. Previous studies have demonstrated the crucial role of ECM reconstruction in the regeneration of the intestine [21], central nervous system [20], and body wall [16] of sea cucumbers. Matrix metalloproteases (MMPs) are enzymes that play integral functions in the degradation of specific ECM components and promote tissue regeneration by facilitating cell proliferation, migration, differentiation and apoptosis [42]. Additionally, tissue inhibitors of metalloproteinases (TIMPs) regulate the degradation of ECM components and tissue remodeling by interacting with MMPs [43]. In a transcriptomic analysis of intestine regeneration in *A. japonicas*, upregulation of all MMPs except *MMP14* was observed, in contrast to our findings during body wall regeneration [21]. During the body wall regeneration process, the downregulation of multiple MMPs and TIMPs suggests a potential reduction in ECM degradation. Furthermore, the differential expression of genes involved in ECM components and ECM-modifying proteins at day-3 and day-7 highlights the significance of ECM reconstruction. These genes likely contribute to the remodeling of connective tissues and the restoration of tissue architecture following injury.

The activation of regeneration-related signaling pathways at the transcript level was observed during the early stage (day-0 and day-3). By day-7 of regeneration, downregulation of *Wnt1*, *Wnt5A*, *FZD2*, *LRP*s and *CTNB* were appeared in the upstream pathway, resulted in the downregulation of *TF7L2*, *Myc*, and *PPARA* in the downstream pathway, subsequently impacting cell proliferation (Fig. 6c). The Wnt/β-catenin signaling pathway is primarily responsible for regulating cell proliferation [44]. However, our findings differ from previous reports regarding the expression levels of this pathway in intestine regeneration [21]. In addition, *YAP1A*, a transcription factor in the Hippo pathway, also exhibited downregulation. The overexpression of YAP in the nucleus promotes cell proliferation through its interaction with β-catenin [45]. In our analysis, the MAPK signaling pathways were found to be enriched with upregulated genes, while the TGF-β signaling pathway was enriched with downregulated genes. The TGF-β pathway regulates various cellular activities, including cell proliferation, apoptosis, and differentiation [46]. The downregulation of *BMP2B*, *SMAD6* and *BAMBI,* as well as the upregulation of TGFR1, was observed, suggesting their involvement in inhibiting cell proliferation during early body wall regeneration. Furthermore, the downregulation of transcription factors *RRAS2* and *MAPK1*, components of the MAPK family, implies their potential role in regulating cell proliferation and differentiation during regeneration stages [47].

Differential expression of the pluripotency factors *Myc*, *Klf2*, *Sox2* and *Oct1* was observed in the transcriptome of *H. leucospilota*. Specifically, *Sox2* showed upregulation, whereas the other genes exhibited downregulation. Results of *IS*H revealed a decrease in the *Klf2*-positive signal in the regenerating body wall connective tissue (Fig. 7a-c), while an increase in the *Sox2*-positive signal was observed in the regenerating body wall coelomic cell layer (Fig. 7d-f). Pluripotent stem cells can be induced from mouse embryonic or adult fibroblasts by introducing Oct3/4, Sox2, c-Myc and Klf4 [48]. Studies have reported the involvement of Myc, SoxB1, and Klf13 genes in intestinal regeneration in sea cucumbers *H. glaberrima* and *A. japonicus* [19, 49]. *Myc* has exhibited differential expression in the central nervous system regeneration of *H. glaberrima* [20]. Differential expression of *Oct4*, *Sox2*, and *c-Myc* has been reported in the regeneration process of the earthworm *Eisenia foetida* [50].

The development-related gene *Hox-B1* displayed upregulated expression in the early phases of body wall regeneration in *H. leucospilota* (Fig. 6d). Following injury, several genes associated with stress response were activated, including *HSP70*, *SAA*, *SODC*, *CATA* and *GST4*. Additionally, apoptosis-related genes such as *CASP7*, *CASP8*, and *BIRC5* exhibited differential expression (Fig. 6d). In addition to regeneration, stress-related antioxidant [51, 52] and apoptosis [29–31, 53] also play crucial roles in responses to the pathogen infection in sea cucumbers. Furthermore, muscle dedifferentiation is observed during the regeneration of visceral organs in echinoderms, including *H. leucospilota* [54]. Furthermore, Histological analysis revealed muscle cell dedifferentiation on day-3 and day-7 (Fig. 2). Transcriptome analysis revealed alterations in gene expression linked to *TBA1A*, *ACT*, *GELS1* and *MYO3B* during the process of body wall regeneration (Fig. 6d). These findings suggest the involvement of cell cytoskeleton formation, which is consistent with previous studies on intestinal regeneration in *A. japonicus* [55].

## Conclusion

An Illumina transcriptome analysis was conducted on *H. leucospilota* at 0-, 3-, and 7-days post-induced fission to examine gene expression patterns during early regeneration. The functional annotation of DEGs not only validates previous reported cellular events [16] but also opens up potential new avenues for future research. The genes implicated in early body wall regeneration can be categorized into four groups: genes associated with extracellular matrix reconstruction, pluripotency factors, signaling pathways, and other genes involved in regeneration. This study enhances our understanding of the molecular mechanisms involved in regeneration in echinoderms, and may also provide insights into the regenerative mechanisms of higher vertebrates.

## Methods

### Animals and artificially induced transverse fission

Healthy *H. leucospilota* were collected from Daya Bay, Shenzhen, Guangdong Province, China. Prior to the experiment, they were acclimated in glass tanks with aerated seawater for a week. Nine sea cucumbers were randomly selected and divided into three groups, with three individuals in each group. By gradually constraining the middle section of the sea cucumbers using rubber bands, they were eventually subjected to induce transverse fission (Fig. 1a). Morphological changes in the regenerating regions of the sea cucumbers after fracture were observed through a stereomicroscope (Fig. 1b). The body wall tissues from the 0-day, 3-day, and 7-day groups after transverse fission, were promptly frozen using liquid nitrogen following dissection.

### RNA extraction and qualification

Total RNA was extracted from the body wall tissue samples of *H. leucospilota* using TRIzol Reagent (Invitrogen, USA). The concentration and purity of the extracted RNA were determined using Nanodrop2000 (Thermo Fisher Scientific Inc., USA), while its integrity was assessed through 1% agarose gel electrophoresis. The RNA Integrity Number (RIN) values were determined using Agilent2100 (Agilent, USA). For the construction of a single library, a minimum of ≥ 1ug of total RNA was required, with a concentration of ≥ 35 ng/μL, OD260/280 ≥ 1.8, and OD260/230 ≥ 1.0.

### Library construction and sequencing

For the sequencing experiment, the Illumina TruSeqTM RNA sample prep Kit method (Illumina Inc., USA) was utilized for library construction, and sequencing was performed using the Illumina Novaseq 6000 sequencing platform (Illumina Inc.). In brief, mRNA was isolated from total RNA using magnetic beads with Oligo (dT). The mRNA was randomly fragmented, and the resulting small fragments of approximately 300 bp were isolated through magnetic bead screening. Subsequently, the double-stranded cDNA was synthesized using mRNA as a template, with the addition of six random base primers, and ligated with an adapter. The sequencing process was then accomplished on the Illumina platform (Illumina Inc.).

### Sequencing data quality control and sequence mapping

To obtain high-quality sequencing data (clean data), the software fastp (https://github.com/OpenGene/fastp) was used to filter out sequencing connector sequences, low-quality read segments, sequences with a high uncertain base information rate (N), and sequences with a length that was too short in the original sequencing data. Subsequently, the clean data (reads) were compared with the *H. leucospilota* reference genome [22] using the software TopHat2 (http://tophat.cbcb.umd.edu/) to obtain mapped data (reads) for further analysis.

### Transcript assembly and functional annotation

The assembled transcripts were compared with known transcripts to obtain new transcripts without annotation information. Then, functional annotation was carried out on these new transcripts. In order to obtain comprehensive gene or transcript annotation information, all the genes and transcripts obtained from transcriptome assembly were compared with various databases. In this case, the databases included NR, Swiss-Prot, Pfam, Clusters of Orthologous Groups of proteins (COG), Gene Ontology (GO), and Kyoto Encyclopedia of Genes and Genomes (KEGG).

### Analysis of differentially expressed genes

To quantitatively analyze the overall expression level of genes, RSEM was utilized and obtain the quantitative index Transcripts Per Million reads (TPM). DESeq2 was used to analyze the differential expression of genes between two groups (*n* = 3). Genes were considered differentially expressed genes (DEGs) with significance levels of adjusted *P* < 0.05 and |log2FC|≥ 1.

### GO and KEGG enrichment analysis of DEGs

The Gene Ontology (GO) enrichment of DEGs was performed using the Goattools Python package (https://github.com/tanghaibao/GOatools). Upon mapping gene IDs to GO terms, *P* < 0.05 were used to determine statistically significant enrichment. Similarly, For the Kyoto Encyclopedia of Genes and Genomes (KEGG)[56, 57] enrichment analysis of DEGs, the Gene IDs were mapped to KEGG pathways, and statistical significance was determined by *P* < 0.05.

### Tissue sectioning and hematoxylin/eosin (HE) staining

The body wall tissues fixed with 4% paraformaldehyde were subjected to a series of steps, including dehydration, clarification, wax dipping and embedding. Subsequently, the wax blocks were sliced at a thickness of 3 μm using a paraffin slicer. The tissue slices were placed on a water bath at 40℃ to flatten, and then delicately transferred onto glass slides. These slides, containing the tissue sections, were then baked in a 60℃ oven. The dewaxed and washed paraffin sections were subsequently stained with hematoxylin and eosin, followed by dehydration and sealing. Finally, the sections were examined under a microscope.

### In situ hybridization

To validate the reliability of the transcriptomic data, *IS*H was performed to conduct cellular localization and relative expression of selected genes. Specifically, the PCR DIG Probe Synthesis Kit (Roche, Switzerland) was used to synthesize DNA probes labeled with digoxigenin (DIG). For *IS*H, body wall tissue samples from sea cucumber were first collected and overnight fixed in a 4% paraformaldehyde fixation solution (Sangon Biotech, China). After fixation, the tissues were dehydrated in gradient ethanol and embedded in paraffin. The wax block was cut into sections of 3 μm in thickness. Subsequently, the sections were immersed in xylene and gradually transferred to ethanol for the purpose of dewaxing. Once dewaxed, the sections were incubated in Proteinase K (20 μg/mL) for 30 min. Endogenous peroxidase was blocked, followed by dropwise addition of prehybridization solution. The sections were then subjected to hybridization by dropwise addition of the probe-containing hybridization solution. Subsequently, the sections were washed sequentially with 2 × SSC, 1 × SSC, and 0.5 × SSC solutions. After that, the sections were undergoing a series of sequential treatments, including blocking with BSA, addition of mouse anti-digoxigenin antibody labeled with horseradish peroxidase (anti-DIG-HRP), and introduction of the 3,3’-Diaminobenzidine (DAB) chromogenic solution. After completion of the DAB color development, the nuclei were stained with Harris Hematoxylin. The sections were dehydrated in a gradient ethanol series and xylene, and finally sealed with neutral resin. The sections were observed under a microscope and photographed for further analysis.

## Data Availability

The datasets presented in this study can be found in NCBI with accession number: PRJNA1007557.

## Abbreviation

CO1A2: Collagen alpha-2(I) chain
CO4A1: Collagen alpha-1(IV) chain
CO6A3: Collagen alpha-3 (VI) chain
GPC1: Glypican-1
FBN1: Fibrillin-1
FBN2: Fibrillin-2
FBN3: Fibrillin-3
FBLN1: Fibulin-1
FBLN2: Fibulin-2
LAMB1: Laminin subunit beta-1
LAMA3: Laminin subunit alpha-3
TSP1: Thrombospondin-1
TSP4: Thrombospondin-4
PRS2: Serine protease 23
PRS3: Serine protease 33
CATD: Cathepsin D
CATL: Cathepsin L
ADAMTS: A disintegrin and metalloproteinase with thrombospondin motifs
MMP: Matrix metalloproteinase
TIMP: Tissue inhibitors of metalloproteinase
FBP1: Fibropellin-1
TENR: Tenascin-R
KLF2: Krüeppel-like factor 2
KLF13: Krüeppel-like factor 13
SOX2: Transcription factor SOX-2
SOXB1: Transcription factor SOX-B1
OCT1 (PO2F1): POU domain, class 2, transcription factor 1;OCT4 (PO5F1): POU domain, class 5, transcription factor 1
WNT1: Protein Wnt-1
WNT5A: Protein Wnt-5a
FZD2: Frizzled2
CTNB: Catenin beta
BAMBI: BMP and activin membrane-bound inhibitor homolog
CSK: Tyrosine-protein kinase CSK
TF7L2: Transcription factor 7-like 2
LRP: Low-density lipoprotein receptor-related protein
PPARA: Peroxisome proliferator-activated receptor alpha
YAP1A: Yes association protein 1-A
TGFR1: TGF-beta receptor type-1
BMP2B: Bone morphogenetic protein 2-B
SMAD6: Mothers against decapentaplegic homolog 6
RRAS2: Ras-related protein R-Ras2
MAPK1: Mitogen-activated protein kinase 1
HXB1: Homeobox protein Hox-B1
Hox: Homeobox protein
BIRC5: Baculoviral IAP repeat-containing protein 5
SAA: Serum amyloid A protein
HSP70: Heat shock 70 kDa protein
SODC: Superoxide dismutase [Cu–Zn]
CATA: Catalase
GST4: Glutathione S-transferase 4
CASP7: Caspase-7
CASP8: Caspase-8
TBA1A: Tubulin alpha-1A chain
ACT: Actin
GELS1: Gelsolin-like protein 1
MYO3B: Myosin-IIIb
ECM: Extracellular matrix
BMPs: Bone morphogenetic proteins
BMI-1: Polycomb complex protein BMI-1
DEGs: Differential expression genes
*IS*H: In situ Hybridization
MCT: Mutable collagenous tissue
RIN: RNA Integrity Number
NR: Non-Redundant Protein Sequence Database
GO: Gene Ontology
KEGG: Kyoto Encyclopedia of Genes and Genomes
COG: Clusters of Orthologous Groups of proteins
RSEM: RNA-seq by expectation maximization
DAB: Diaminobenzidine
